# Morphometrics of *Xenopus laevis* Kept as Laboratory Animals

**DOI:** 10.3390/ani12212986

**Published:** 2022-10-30

**Authors:** Linda F. Böswald, Dana Matzek, Helen Mohr, Ellen Kienzle, Bastian Popper

**Affiliations:** 1 Chair for Animal Nutrition and Dietetics, Ludwig-Maximilians-Universität München, Schönleutnerstr. 8, 85764 Oberschleißheim, Germany; 2 Biomedical Center, Core Facility Animal Models, Faculty of Medicine, Ludwig-Maximilians-Universität München, Großhaderner Straße 9, 82152 Martinsried, Germany

**Keywords:** amphibian, African clawed frog, body condition score

## Abstract

**Simple Summary:**

Body conditions can be indicators of nutritional status and health in animals. Thus, it is important to monitor body conditions. In laboratory animals, this is of special interest during experiments, on the one hand, to evaluate the stress the animal is exposed to, and on the other hand, the recordings of these parameters are important in order to provide the animals with appropriate husbandry conditions in their lives and metabolic needs. For African clawed frogs, *Xenopus laevis*, as amphibians, the use of body condition scores or indices has not been implemented as yet. In the present study, adult male and female *Xenopus laevis* were weighed and several measurements were taken from standardized photographs to establish tools for body condition evaluations in this species. Such morphometric data can serve as reference data for body condition evaluations.

**Abstract:**

Morphometric data that provide information on body conditions can be used to monitor the health and well-being of animals. In laboratory animals, they can help to evaluate the stress due to experiments or treatments, following the 3R principles. The aim of the present study was to obtain morphometric data of male and female African clawed frogs, *Xenopus laevis*, as the bases for body condition evaluations. Adult frogs (*n* = 198) were weighed and standardized photographs were taken. The photographs were used to determine several measurements (length, cranial width, caudal width, thigh width). In addition, a triangle was drawn to outline each frog’s simplified body form, and the triangle surface was calculated. In conclusion, the triangle surface drawn on the dorsal plane of each frog correlated with the body weight of the females. There were significant differences between the body weights and sizes of male and female frogs, with males being smaller (*p* < 0.001). Based on the morphometric data, females could be assigned to five groups in which an assessment of the animal’s well-being is feasible.

## 1. Introduction

In laboratory animals, body condition evaluations are used as sensitive measures of animal welfare/their health statuses [1,2,3]; moreover, they are easy and non-invasive to obtain. The visual assessment of an animal (according to a standardized scoring system that takes into account fat reserves) is important, because body weight alone may be misleading in the case of, e.g., ascites, tumor growth, or organomegaly [3]. Unintentional loss of body mass can indicate stress or pain, which is important to estimate the overall severity of an experiment. In terms of the 3Rs [4], body condition evaluations contribute to animal welfare and, thus, refinement of trial conditions. The evaluation may even be used among the criteria used for when a trial should end for an individual, indicating severe weight loss.

There are different ways to evaluate body conditions. In mammalian species, a species-specific body condition score (BCS) is commonly used to objectively assess nutritional status. Validated systems exist for cats and dogs [5,6], cattle [7], pigs [8,9], horses [10], and several exotic species [11,12,13,14]. Body condition scoring under practical conditions in veterinary practice is usually performed via visual and—where possible—palpatory evaluations, giving estimates about body composition and body fat mass [5]. For horses, morphometric measurements in combination with body weight can be calculated to evaluate body conditions [10].

While BCS systems that rely on breast muscle development exist for poultry [15], for other birds, the use of morphometrics is more common [16]. Morphometric data provide information about the dimensions of the body, changing over time within one individual or differing between individuals of a species. In many species—especially non-domestic species, including marine mammals, birds, reptiles, fish, and amphibians—such data are important to describe a population and evaluate the individual’s body conditions [17,18,19,20,21], which can be important for species conservation. Even though several body condition indices are calculated from morphometric data, the direct use of morphometric data for evaluation has been recommended [22].

In amphibians, body condition scoring is not performed in a similar way as in mammalian species. Since amphibians deposit body fat intracoelomically rather than subcutaneously [23,24], the adspectory assessment of body fat mass (as usual) in mammals is not translatable. One visual score based on the form of defined body regions has been validated for mountain chicken frogs (*Leptodactylus fallax*), which is a terrestrial species belonging to the order Anura [23]. In other amphibian species, morphometrics are used to describe populations [17,25,26,27,28,29,30], with the snout–vent–length (SVL) or snout–urostyle-length (SUL), respectively, being the most important parameter. Disi and Amr [17] list 17 measurements taken from the amphibian species, including, e.g., toe length and eye diameter. Taking all of these measurements was, however, only possible in dead specimens. Body weight is seldom recorded in wildlife studies [29]. Several body condition indices exist for population descriptions of free-ranging amphibian species [25]. An example is the yellow-bellied toad (*Bombina variegata*), for which three indices were compared, with the residual index being recommended for wildlife population monitoring [25]. However, the concept of these indices is to describe the population in conservational research, but not to follow up on the development of an individual [29], as would be the aim of, e.g., health monitoring in captive animals. Thus, the indices used in wildlife population studies do not meet the aim of the present study.

The aquatic amphibian species *Xenopus laevis*, the African clawed frog, has been used as a laboratory animal for over a century with different research aims [31]. Feeding practices vary between facilities [32]. In this context, for husbandry, as well as for the use of *Xenopus* frogs in an experiment, a tool to evaluate the body conditions of the frogs would be useful. It could give an indication of the adequacy of the feed and the amount of feed per tank by documenting the body condition over time. During an experiment, recording the body condition could be part of the standard protocol (as in mammals) as an indicator of health and wellbeing. Due to *Xenopus laevis* having a rather different body shape than the species with established body condition indices, the value of translating those indices was not believed to be successful.

Morphometric data are important as reference data on the size and shape of a population of animals, as described above for non-domestic species. These measurements can be used as reference values in themselves and could be the bases for the development of a classification system for body condition, distinguishing between “average” animals and those below or above the average values. The aim of the present study was, therefore, to generate basic morphometric data of adult, clinically healthy, male and female *Xenopus laevis* frogs for further classification of body conditions.

## 2. Materials and Methods

### 2.1. Animals and Housing

Ethical approval was obtained from the Government of Upper Bavaria, Az. 03-19-064. All experiments were performed in accordance with German and European animal welfare legislations. In the spirit of reducing animal numbers in research projects, no animals were purchased for this study, but rather animals already housed in the Core facility animal models (Biomedical Center, LMU München) were used. All frogs were kept in the facility for more than 3 months at the time of the study. Clinically healthy, adult *Xenopus laevis* specimens (age range: 6–11 years) in maintenance state were used for the study. They were housed in groups of 10–15 animals per tank (87.3 liters) in a semi-closed system containing UV radiation, water filtration, and an automated water conditioning apparatus (Aqua Schwarz, Goettingen, Germany). The dimensions of the tanks were: length 99.5 cm, width 58.5 cm, and height 25 cm (Aqua Schwarz, Germany). Each tank was enriched with a dark shelter for the animals to hide under. Water temperature, pH value, and water conductivity (Sv) were recorded daily and set to standard conditions of ~18°, pH~8, and Sv~542. The water was changed partially three times per day by an automated system. The light cycle was adjusted to 12 h light and 12 h dark periods. Health monitoring was performed quarterly, including analysis of water parameters (ph, Sv,) and necropsy, bacteriology, and mycology of 1–2 animals in the same water circuit (monitoring parameters matching the required values of the facility). The frogs were fed twice a week with 15 g of fish feed/tank/meal (Teichsticks Premium, Interquell, Wehringen, Germany).

The study cohort was composed of *Xenopus laevis* frogs (40 males and 198 females).

### 2.2. Body Condition Parameters

**Figure 1:**
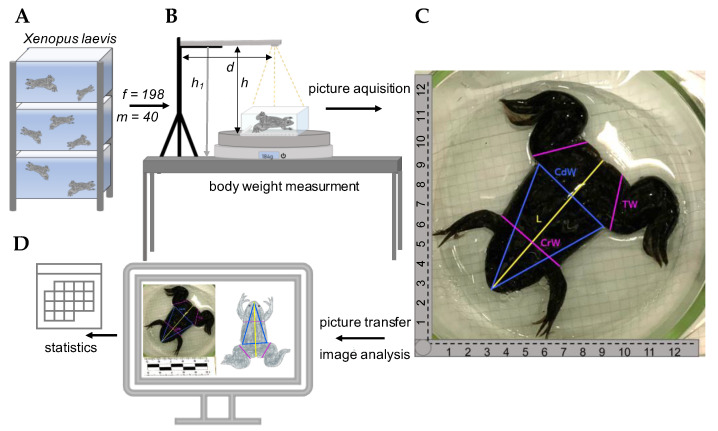
(**A**) *Xenopus laevis* frogs were housed in groups of 10–15 animals. In total 198 males (m) and 40 females (f) were used to establish the method. (**B**) Camera set-up. The tablet was mounted on the tripod at a fixed distance (*d*) and height of 49 cm (*h*_1_) above the table, resulting in a 44 cm distance between the tablet and the ground of the basin (*h*), where the frogs were placed. Body weight was measured for each frog in parallel to the image acquisition. (**C**) Lines showing all the measurements that were taken from each frog (cranial width CrW, caudal width CdW, snout–vent length L, thigh width TW). The metric scale was used for the calculation of actual distances from the photograph. (**D**) GIMP© image analysis software was used to measure distances drawn on the frogs’ dorsal planes.

The frogs were transferred from their tanks into another tank filled with water from the same circulation system to avoid any distress. One by one, they were put into a glass basin (ca. 15 × 20 cm) filled with a small amount of water (just covering the ground of the basin and accounted for by setting the tare weight) located on a scale 10 × 10 cm (LS10, ATG Kriminaltechnik GmBH, Berlin, Germany). The body weight (BW in grams) of the frog was determined by a digital scale (900-8641, Henry Schein, Berlin, Germany) and a photograph was taken of the frog in the basin from above. For the photographs, a tripod was set up next to the scale, holding a tablet with an integrated camera (iPad Pro 2009, 12 Megapixel, Apple, Cupertino, CA, USA) at a fixed height of 49 cm (*h*_1_ = 49 cm; see Figure 1). After the photograph was taken, the frogs were put back into their original tank. The water basin on the scale was cleaned after each tank.

The photographs of the frogs were used for measurements since prolonged handling and positioning of the limbs would have been too stressful for the animals. For measurements, the software GIMP© was used (GNU Image Manipulation Program, version 2.10.30). The metric scale on the photographs allowed for converting the distances in the photograph into actual distances (cm). The following measurements were taken from each frog (modified after Disi and Amr [17], see Figure 1C):

- Snout–vent length (L): length from the tip of the snout to the posterior margin of the cloaca (cm).
- Cranial width (CrW): width from the left to the right side, measured directly behind the forelimbs (cm).
- Caudal width (CdW): width from the left to the right side, measured directly in front of the hindlimbs (cm).
- Thigh width (TW): the thigh width was measured at the broadest site (cm).
- Triangle surface (TS): a triangle was formed from the tip of the snout to the caudal width and the surface was calculated (TS = 0.5·CdW·height of the triangle; cm^2^).

For comparison with other studies on amphibians, the following index was calculated [25]:

- Relative mass condition index W_R_ = 100∙BW/BWs with BWs being the BW predicted by the linear correlation between BW and L in a log_10_ transformation.

### 2.3. Statistics

Statistics were conducted using SigmaPlot 14.0 (Systat Software, San Jose, CA, USA). Body parameters were compared between the sex groups via a t-test in case of normal distribution of data and via the Mann–Whitney rank sum test if normality testing failed. The significance level was set to α < 0.05.

Regression models were calculated for several measurements. For the correlation of body weight and triangle surface, the slopes of the regression lines of female and male frogs were compared with the test of Ho in BiAS for Windows (Version 11.05, 2017, epsilon Verlag, Frankfurt, Germany).

## 3. Results

**Figure 2:**
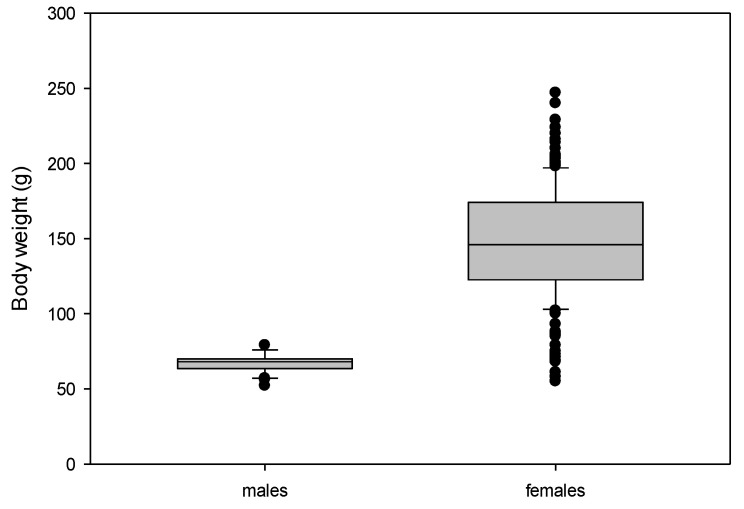
Body weight distribution in adult males (*n* = 40) and females (*n* = 198). *Xenopus laevis* (α < 0.001).

**Figure 3:**
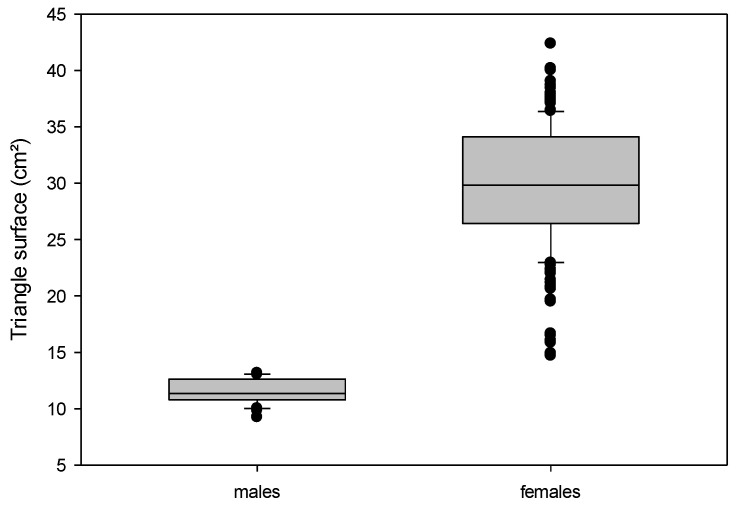
Comparison of the triangle surface (cm^2^) in adult males (*n* = 40) and females (*n* = 198). *Xenopus laevis* (α < 0.001).

The female frogs had significantly higher body weights than males, as well as higher variations of this parameter (range 55–247 g in females, 52–79 g in males; see Table 1; Figure 2). The body measurements were also significantly higher for the female frogs, in particular, the lengths of the females ranged much higher (females 8.4–14 cm, compared to 7.1–8.3 cm in males). Female frogs had significantly higher triangle surfaces than the males (*p* < 0.001; Figure 3). In some frogs (8 females, 2 males), it was not possible to measure the thigh width due to their sitting positions with overlap between the thigh and body or the rest of the limb.

**Figure 4:**
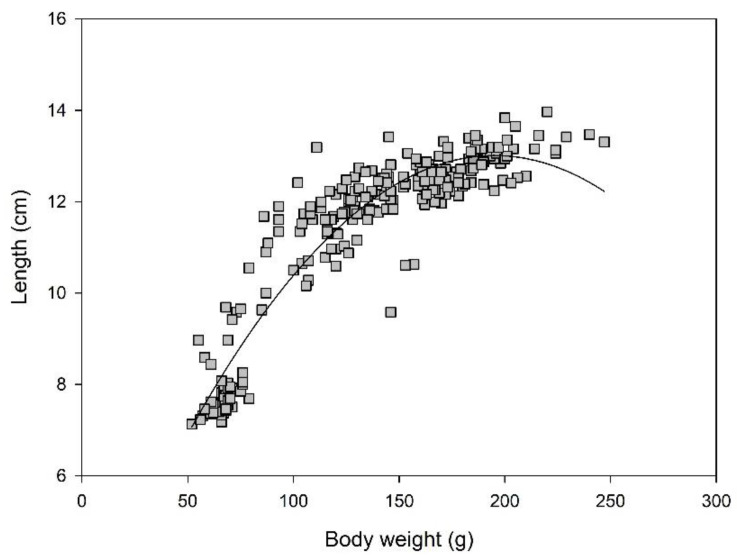
Exponential correlation between body weight (*x*-axis) and length (*y*-axis) of adult *Xenopus laevis* frogs (*n* = 238, y = −0.0003x^2^ + 0.1132x + 1.9692; R^2^ = 0.87).

The ratio of body weight: length was significantly higher in the female frogs (12.16 ± 2.45 vs. 8.75 ± 0.61; *p* < 0.001). For frogs of both sexes combined, body weight and length were correlated strongly in an exponential function (R^2^ = 0.91; Figure 4). The slope starts to decline with a body weight of ca. 140–150 g, after which, a kind of plateau is reached. This could also be described in a broken line model with a linear relationship up to a BW of <140 g (y = 0.064x + 3.93; R^2^ = 0.83) and a nearly flat line for the animals with BW ≥ 140 g (y = 0.04x + 10.11; R^2^ = 0.32). Thigh width proved to be difficult to measure because of variations in the limb positions of the frogs. Therefore, it was not used for further evaluation or regression analysis.

**Figure 5:**
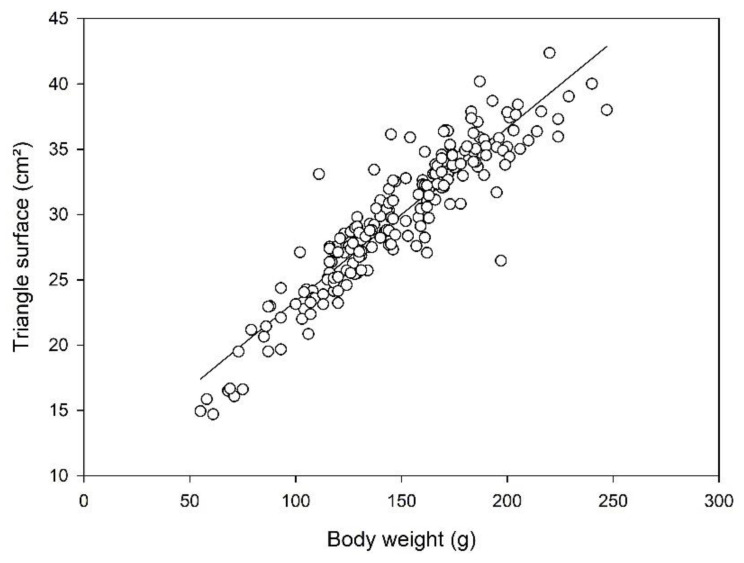
Strong linear correlation between body weight (*x*-axis) and triangular surface (*y*-axis) of female adult *Xenopus laevis* frogs (*n* = 198; y = 0.13x + 10.11; R^2^ = 0.85).

In females, there was a strong linear correlation between the triangular surface (*x*-axis) and the body weight (*y*-axis; y = 0.13x + 10.11, R^2^ = 0.91; Figure 5). This correlation was weak in the male frogs (y = 0.10x + 4.83; R^2^ = 0.31; Supplementary Figure S1), but the slopes of the regression lines did not differ significantly between the sexes (*p* = 0.187).

**Figure 6:**
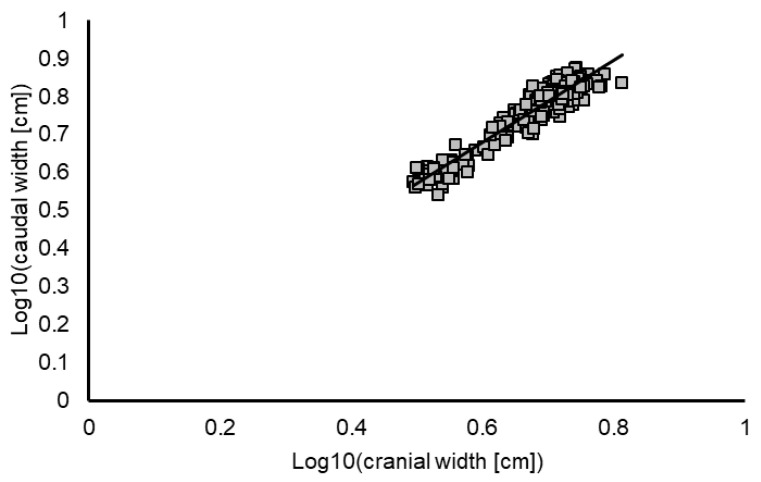
Strong linear correlation between log_10_ transformed data on cranial width (*x*-axis) and caudal with (*y*-axis) of *Xenopus laevis* frogs of both sexes (*n* = 238; y = 1.08x + 0.03; R^2^ = 0.91).

There was also a strong linear correlation between the log-transformed cranial and caudal width measurements (Figure 6; R^2^ = 0.91). The quotient of caudal to cranial width was significantly higher in the female frogs than in the males (means ± SD: 1.23 ± 0.07; 1.17 ± 0.06; *p* < 0.001).

**Figure 7:**
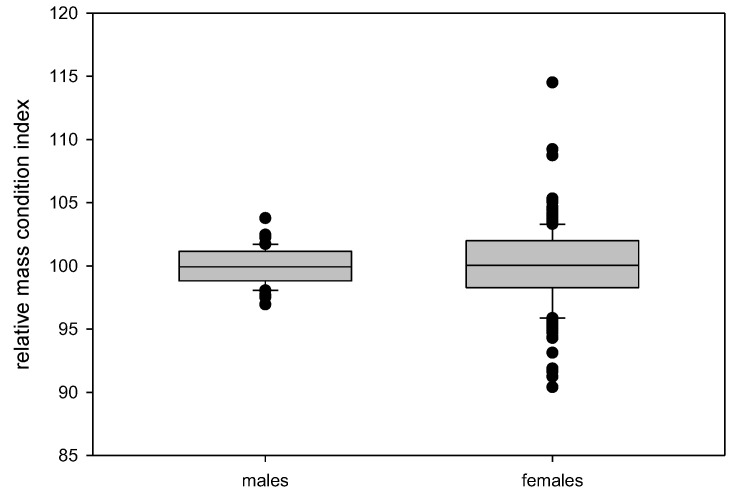
Relative mass condition index W_R_ calculated separately in male (*n* = 40) and female (*n* = 198) *Xenopus laevis* frogs.

The relative mass condition index W_R_ was calculated separately for each sex because of the differences in body weight and morphology observed in the morphometric parameters described above. The females showed a considerably higher variation of this index (Figure 7). The triangle surface and W_R_ did not correlate well in females (y = 0.23x + 93.00; R^2^ = 0.17) and males (y = 0.05x + 99.46; R^2^ = 0.001).

**Figure 8:**
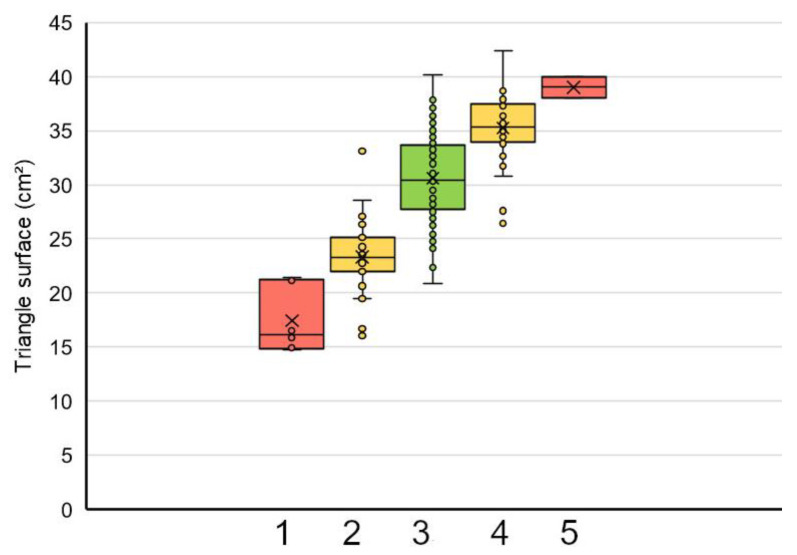
Triangle surface (cm^2^) scaled according to the groups calculated from body weight: length (group 1–5) (female frogs, *n* = 198). There were significant differences between groups 1 and 2 on the one hand and groups 3, 4, and 5 on the other hand (*p* < 0.001), except for 4 and 5 (*p* = 0.151). The color coding indicates average (yellow) and higher deviations (red) from the average (green).

**Figure 9:**
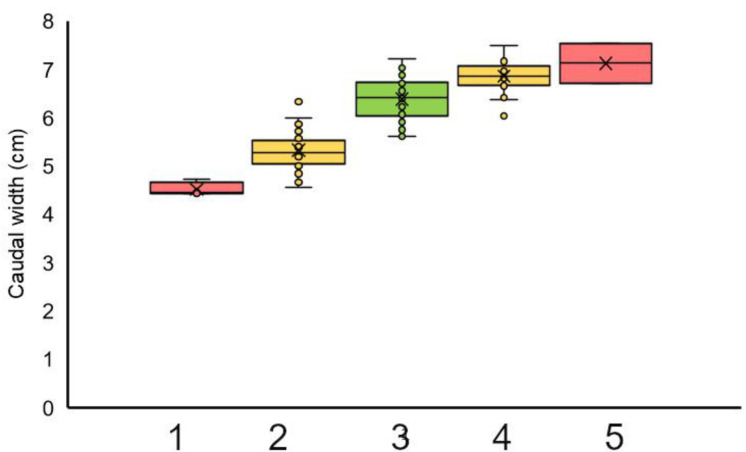
Caudal width (cm) scaled according to the groups calculated from body weight: length (group 1–5) (female frogs, *n* = 198). Means ± SD: 1 (*n* = 4): 4.51 ± 0.14; 2 (*n* = 33): 5.33 ± 0.44. The color coding indicates average (yellow) and higher deviations (red) from the average (green).

The quotient of body weight: length was used to group data in the cohort of female frogs: group 1 < mean − 2∙SD; mean − 2∙SD < group 2 < mean − SD; group 3 = mean ± 1∙SD; mean + SD < group 4 < mean + 2∙SD; Group 4 > mean + 2∙SD (see Table 2). The triangle surface could be scaled according to these groups (Figure 8). The triangle surface and caudal width (Figure 9) were significantly higher in groups 3, 4, and 5 than in 1 and 2 (*p* < 0.001).

## 4. Discussion

The purpose of this study was to evaluate morphometrics on adult male and female *Xenopus laevis* frogs kept as laboratory animals to assess body conditions in accordance with the adspectory and palpatory BCS methods used for other laboratory animals. To date, no score or index for body conditions has been developed specifically for this *Xenopus laevis*. Thus, the morphometric dataset generated in this study should allow a standardized assessment of the frogs in the context of veterinary stock management, e.g., monitoring of the health status and stress assessment.

In research on free-ranging amphibian species, morphometrics are frequently used to describe populations [17,27,29,33,34]. Handling the animals to correctly take several measurements would necessitate fixation and take time, which means distress for the frogs. This is not feasible in the routine assessment of the frogs´ health status in a laboratory animal facility. In this study, morphometric data were generated using standardized photographs of the dorsal plane of adult *Xenopus laevis* frogs. The photographs were taken from above, capturing the frogs sitting relaxed in a glass basin filled with a small amount of water. This method allowed obtaining several measurements at a time without handling or fixating the frogs for a long time. Taking the measurements from photographs may leave a small error in translation via a metric scale. Given the fixed camera setup, this potential error was the same for all photographs and, thus, did not influence the comparison between the frogs of the study. On the contrary, the evaluation of the parameters with the integration of an image processing program allows the standardized collection of the measured variables without the additional error susceptibility by the examiner. Camera-based and automated methods are also used for BCS assessment in livestock and allow the detection of changes that might have escaped the observer’s gaze [35]. In addition to measurements, the body weights of the frogs were determined, which is not common in wildlife population studies but gives important information.

There was a strong exponential correlation between body weight and length (R^2^ = 0.87), indicating an overall higher body condition in the frogs with higher body lengths. The cut-off seems to be approximately 140 g BW or a length of approximately 12 cm, after which a plateau is reached. This can indicate a BW gain above the capacity for growth, i.e., the development of body fat storage. In terms of body condition, it may be assumed that this can be evaluated as the development of the animal becoming overweight in a maintenance metabolic state. Both being underweight and overweight can have negative consequences for health and reproductive performance. To differentiate between the gain of additional fat or muscle mass, a body composition analysis (in vivo DEXA scans or post-mortem Weende analysis) would be necessary.

The population of adult *Xenopus laevis* used for this study showed distinct sexual dimorphisms in all measurements, with females being significantly larger than males. In addition to being larger than the males, the female frogs showed considerably higher variations in body weight and size. The relative mass condition index W_R_ also showed a difference in the distribution between the sexes (Figure 7). This sexual dimorphism of size is known in many amphibian species, including *Xenopus* [36]. It is speculated that in species where the males are not fighting for mating success, size is not a factor for genetic selection and, thus, the males are not larger than the females [37]. In wild populations, the size dimorphism might also reflect the age structure, with females reaching a higher age [38]. Whether the latter assumption holds true for a captive setting under standardized conditions seems questionable. In addition to the dimorphism, as seen in the measurements, there is evidence that in anurans, specifically *Xenopus allofraseri*, females have a higher basal metabolic rate [39]. For both reasons, female and male frogs´ body conditions cannot be evaluated with one set of data.

In the study population of male frogs, morphometric data were rather homogenous without obvious outliers (Table 1). To define groups over a broader range of body conditions, it is necessary to include data from individuals differing markedly from the average in both directions. Thus, a further analysis for the classification of body condition groups was not possible for the males. The morphometric data obtained from the male population given in Table 1 can be used as reference data for healthy, adult male *Xenopus laevis* frogs under similar laboratory settings. To define values for below- and above-average morphometrics, a more heterogeneous population would be necessary.

Since clawed frogs do not show externally visible fat depositions or clearly palpable bone structures [24] that allow conclusions regarding BCS, we used well-established morphometrics that are already commonly used in amphibians to compare individuals within a species [23]. At this point, we expanded the options for assessment to include new metrics—the triangular surface—on the dorsal body side of clawed frogs and compared the parameter with known measured values, such as the body length and animal weight.

The triangle surface and relative mass condition index [25] did not correlate well (in females: relative mass condition index = 0.23∙TS + 92.99; R^2^ = 0.17). This may be due to the rather high variations in length and body weight of the female *Xenopus laevis*, so the body condition was not sufficiently described by body weight. For species with fewer variations in age and, therefore, size [38], such as wildlife populations, the relative mass condition index can be suitable.

In female *Xenopus laevis*, body weight and calculated triangle surface were strongly correlated in linear regression (R^2^ = 0.85). This means that increasing body weight led not only to a longer body but also to a larger and “broader” abdomen, as seen from above and expressed by the surface of the fictional triangle drawn in the silhouette. The triangular area takes into account that the animals, especially in frequently used deep holding tanks [32], can only be inspected from the water surface. The additional evaluation of measurements when viewing the frog from a lateral angle would necessitate repositioning and, thus, more handling and potential distress for the animals. Thus, the evaluation of body shape in a standardized setting is preferable to evaluating the frogs in spontaneous movement in the tanks, due to the influence of movement on their positioning. Log_10_ transformed data of cranial and caudal widths also showed a strong linear correlation (R^2^ = 0.91), supporting the consideration of a change in the abdominal shape with increasing body weight. It must be taken into account that this method can only be used for healthy animals and likely needs to be adapted for deviations in body shape, for example, due to surgical interventions or hormone stimulation. An evaluation of the method in animal experiments should be provided in further studies.

Since the triangle surface is a measurement of both the length and shape, this parameter was used to classify the female frogs into five groups, choosing the scale of five in the manner of several mammalian body condition scores [40]. There were significant differences between the five groups. Visual differentiation between frogs of the groups according to the standardized pictures may, however, be challenging. In addition, the groups deviating most from the mean (groups 1 and 5) consisted of only four and two animals, respectively.

With the method and the digital acquisition of the measuring points by integration of the measuring parameters into an image processing program, there is the possibility of linking the morphometric data (in the long term) with the animals; this is a point of view that does justice to the long-life expectancies of the animals and contributes substantially to the improvement of the housing conditions, stocking intensities, feeding protocols, and breeding in the sense of the 3Rs and animal welfare in general.

## 5. Conclusions

With the dataset on female and male *Xenopus laevis* frogs, data on body weight and several body measurements could be generated. There is a distinct sexual dimorphism with female frogs being significantly larger and heavier. In female frogs, body weight and length were strongly correlated. The triangular surface measurement provides a non-invasive and reliable morphometric method to evaluate adult African clawed frogs, following the body condition rating systems of other species. Further work must show whether the morphometric elevation on the carcass introduced in this work also allows for reliable assessments of animal welfare under experimental conditions.

## Figures and Tables

**Table 1:** Body weight and measurements of adult *Xenopus laevis* grouped according to sex.

	Females						Males						
	*n*	Mean ± SD	Range	Median	25% Q	75% Q	*n*	Mean ± SD	Range	Median	25% Q	75% Q	*p*
BW (g)	198	148.8 ± 37.8	55.00–145.00	146.00	122.50	174.25	40	67.0 ± 6.0	52.00–79.00	68.00	63.50	70.22	<0.001
L (cm)	198	12.1 ± 1.0	8.44–13.97	12.31	11.74	12.71	40	7.7 ± 0.3	7.13–8.26	7.67	7.41	7.92	<0.001
CrW (cm)	198	5.1 ± 0.5	3.6–6.50	5.13	4.81	5.42	40	3.4 ± 0.2	3.13–3.79	3.41	3.28	3.53	<0.001
CdW (cm)	198	6.2 ± 0.7	4.44–7.53	6.37	5.81	6.74	40	4.0 ± 0.2	3.49–4.31	4.02	3.86	4.10	<0.001
TS (cm^2^)	198	29.8 ± 5.4	14.70–42.37	29.83	26.44	34.11	40	11.6 ± 1.1	9.25–13.15	11.37	10.79	12.63	<0.001
TW (cm)	190	3.4 ± 0.3	2.23–4.03	3.37	3.21	3.55	38	2.3 ± 0.2	1.97–2.56	2.31	2.18	2.41	<0.001

BW = body weight, L = length, CrW = cranial width, CdW = caudal width, TS = triangle surface, TW = thigh width, SD = standard deviation, Q = quartile, α < 0.05, *n* = number of animals.

**Table 2:** Description of the body condition groups of female *Xenopus laevis*, as grouped by the quotient of body weight by length.

Group	BW/L Range [g/cm]	*n*	TS [cm^2^]	CdW [cm]
1	<7.26	4	15.50 ^a^ ± 0.83	4.51 ^a^ ± 0.14
2	7.26–10.15	33	23.19 ^a^ ± 3.51	5.33 ^a^ ± 0.44
3	10.16–14.61	129	30.59 ^b^ ± 3.70	6.38 ^b^ ± 0.44
4	14.62–17.5	30	35.23 ^b^ ± 3.34	6.85 ^b^ ± 0.31
5	>17.5	2	39.01 ^b^ ± 1.42	7.13 ^b^ ± 0.57

BW = body weight, L = length, CdW = caudal width, TS = triangle surface, *n* = number of animals. Means in a column sharing the same superscript letter do not differ significantly (α < 0.05).

## Data Availability

The full datasets can be made available by the authors upon reasonable request.

## Supplementary Materials

The following supporting information can be downloaded at: https://www.mdpi.com/article/10.3390/ani12212986/s1, Figure S1: Correlation between body weight and triangle surface in male *Xenopus laevis* frogs.
