# Supplementary material for: Morphometrics of Xenopus laevis Kept as Laboratory Animals
Source: Animals (Basel). 2022 Oct 30;12(21):2986. doi: 10.3390/ani12212986 (PMC9653714; doi:10.3390/ani12212986)
Supplement: Supplementary file 1 [file animals-12-02986-s001.zip › animals-1999598-supplementary.pdf]

Supplementary Figure S1

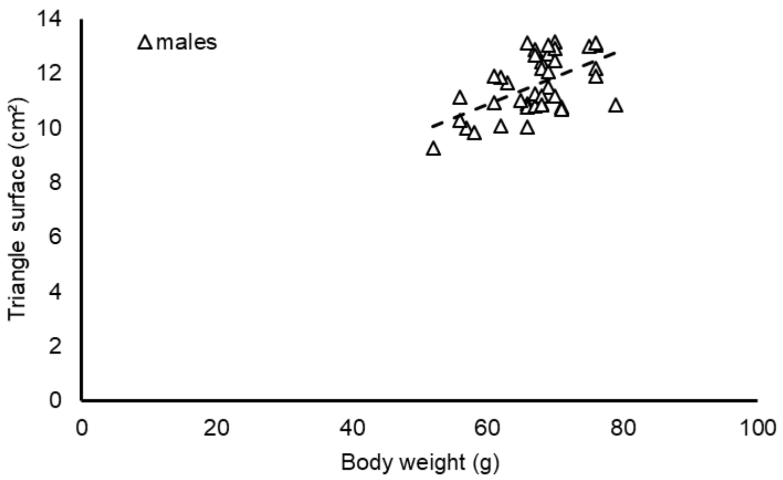

Correlation between body weight (g) and triangle surface (cm^2) in male *Xenopus laevis* frogs (n = 40; y = 0.10 x + 4.83; R^2 = 0.31).
